# Melatonin Modulates Astrocyte Inflammatory Response and Nrf2/SIRT1 Signaling Pathways in Adult Rat Cortical Cultures

**DOI:** 10.3390/biomedicines13122967

**Published:** 2025-12-02

**Authors:** Ester Rezena, Matheus Sinhorelli Cioccari, Aline Daniel Moreira de Moraes, Giancarlo Tomazzoni de Oliveira, Vanessa-Fernanda Da Silva, Izaviany Schmitz, Guilhian Leipnitz, Carlos-Alberto Gonçalves, Carmem Gottfried, Larissa Daniele Bobermin, André Quincozes-Santos

**Affiliations:** 1 Programa de Pós-Graduação em Ciências Biológicas: Bioquímica, Instituto de Ciências Básicas da Saúde, Universidade Federal do Rio Grande do Sul, Porto Alegre 90035-003, RS, Brazil; estercarstairs@outlook.com (E.R.); mathcioccari@gmail.com (M.S.C.); giantomazzoni1@gmail.com (G.T.d.O.); vanessa.fernanda@ufrgs.br (V.-F.D.S.); izaviany.schmitz@ufrgs.br (I.S.); guilhian@ufrgs.br (G.L.); casg@ufrgs.br (C.-A.G.); cgottfried@ufrgs.br (C.G.); larissa.bobermin@ufrgs.br (L.D.B.); 2 Departamento de Bioquímica, Instituto de Ciências Básicas da Saúde, Universidade Federal do Rio Grande do Sul, Porto Alegre 90035-003, RS, Brazil; linemoraesa1@gmail.com; 3 Programa de Pós-Graduação em Neurociências, Instituto de Ciências Básicas da Saúde, Universidade Federal do Rio Grande do Sul, Porto Alegre 90035-003, RS, Brazil; 4 Programa de Pós-Graduação em Ciências Biológicas: Fisiologia, Instituto de Ciências Básicas da Saúde, Universidade Federal do Rio Grande do Sul, Porto Alegre 90035-003, RS, Brazil; 5 Instituto Nacional de Ciência e Tecnologia em Neuroimunomodulação, INCT-NIM, IOC, Fiocruz, Rio de Janeiro 21040-900, RJ, Brazil

**Keywords:** astrocytes, cerebral cortex, glioprotection, melatonin

## Abstract

**Background/Objectives:** The cerebral cortex is critical for neurological functions that are strongly affected by the aging process. Astrocytes play a central role in maintaining neurotransmitter balance and regulating antioxidant and anti-inflammatory responses, but these physiological functions may also decline with age. This study aimed to investigate the effects of melatonin, a molecule with known antioxidant, anti-inflammatory and neuroprotective properties, on astrocytes of mature cortical tissue obtained from adult Wistar rats. **Methods:** Primary cortical astrocyte cultures were obtained from neonatal and 90-day-old Wistar rats and treated with melatonin (300 µM for 24 h). We assessed cell viability and metabolism (MTT and extracellular lactate levels), glutamine synthetase (GS) activity, glutathione (GSH) content, release of cytokines, and the expression of genes and proteins associated with oxidative stress and inflammation by RT-qPCR and Western blotting. **Results:** Melatonin did not affect cell viability or lactate production. Moreover, there were no changes in GS activity, a key enzyme in glutamate metabolism, or in GSH levels, an antioxidant defense molecule synthesized by astrocytes. However, melatonin significantly reduced the expression of the nuclear factor *NFκB*, cyclooxygenase 2 (*COX-2*), and inducible nitric oxide synthase (*iNOS*), while increasing interleukin 6 and 10 levels. Melatonin also upregulated the gene expression of the transcriptional factors *Nrf2* and sirtuin 1 (*SIRT1*) and downregulated AMP-activated protein kinase (*AMPK*) and peroxisome proliferator-activated receptor gamma coactivator-1 alpha (*PGC-1α*), while PGC-1α protein levels remained unchanged. A complementary analysis of astrocytes obtained from neonatal rats showed that melatonin did not change metabolic or redox parameters under basal conditions. **Conclusions:** Melatonin exerted anti-inflammatory effects on adult astrocyte cultures, likely through modulation of protective signaling pathways, such as Nrf2/SIRT1. These findings highlight the potential role of melatonin in preserving astrocytic function and mitigating age-related neuroinflammatory processes.

## 1. Introduction

Melatonin (N-acetyl-5-methoxytryptamine) is a hormone that is involved in the circadian rhythm [1,2]. Its availability declines with age, and this change is associated with neurological disorders [3,4]. In this context, melatonin, particularly in supraphysiological concentrations, has potential therapeutic effects in the treatment of inflammatory and oxidative damage, commonly present in these diseases [5]. Melatonin can act as an indirect activator of antioxidant enzymes and directly through the electron donation of its aromatic indole ring, contributing to redox balance by increasing the efficiency of ATP synthesis and decreasing electron leakage [6]. Moreover, melatonin dynamically modulates immune responses and suppresses excessive pro-inflammatory stimuli by inhibiting prostaglandins and regulating the expression and activity of cytokines and transcription factors [5]. 

The central nervous system (CNS) is a crucial target of melatonin-induced responses, given its interaction with specific receptors located in the brain [1,3,4,7]. With regard to the aging process, melatonin is able to modulate interleukin-1β (IL-1β) activity, as well as tumor necrosis factor α (TNF-α), age-dependently, in the whole hippocampus and cortex brain tissue of aged rodents [8]. In addition, this is particularly important in neuropathological conditions driven by mechanisms such as inflammation, oxidative stress, and altered signal transduction pathways [9,10]. These processes directly involve glial cells, especially astrocytes, which are key elements linking these molecular alterations to broader neural dysfunction [11]. The contributions of cortical astrocytes are critical for understanding neurological and neurodegenerative disorders, because the cortex is a fundamental integrative region for sensory, motor, and decision-making functions [12,13]. In addition, astrocytes play crucial roles in maintaining neural networks and supporting typical CNS functions [11]. These include the glutamate–glutamine cycle, trophic support, blood–brain barrier formation, and involvement in inflammatory and oxidant responses [11,14]. Therefore, these cells are crucial in the study of the main challenges present in most brain pathologies: inflammation, oxidative stress and related signaling pathways. 

Although melatonin is widely recognized for its antioxidant and anti-inflammatory actions in the central nervous system, most evidence has focused primarily on neuronal cells, while its direct effects on astrocytes remain insufficiently characterized. This gap is particularly relevant because astrocytes are dynamic cells with intrinsic protective properties. In this context, glioprotection can be defined as glial responses in both physiological and pathological conditions, by which they can protect themselves as well as neuronal cells, resulting in an overall improvement of the CNS functioning [11]. These protective properties can be positively modulated by molecules such as resveratrol, isoflavones, sulforaphane, and melatonin [15,16,17,18]. However, the studies that have investigated melatonin in astrocytes have predominantly used cultures derived from neonatal rodents. These immature cells display an increased plasticity and do not fully represent the physiological, metabolic, and transcriptional maturation characteristics of adult astrocytes [14,19,20]. Together, these limitations support the need to determine whether melatonin modulates key functional parameters in mature astrocytes. In this context, the present study examines the effects of melatonin on inflammatory, metabolic, and redox pathways in astrocytes derived from adult Wistar rats, providing insights into its potential glioprotective actions in a more physiologically representative experimental model. 

## 2. Materials and Methods

### 2.1. Animals

Adult (90 days old) and neonatal (1 to 3 days old) male Wistar rats were obtained from the Center for Reproduction and Experimentation of Laboratory Animals (CREAL, UFRGS, Porto Alegre, RS, Brazil) and maintained in a controlled environment (12 h light/12 h dark cycle; 2261 µC; ad libitum access to food and water). Animal experiments were performed in accordance with the NIH Guide for the Care and Use of Laboratory Animals and were approved by the Animal Care and Use Committee of the Federal University of Rio Grande do Sul (process number 42858).

### 2.2. Primary Culture of Astrocytes and Melatonin Treatment 

Cortical astrocyte cultures from adult rodents (90 days old) were performed as previously described [14]. The animals were anesthetized by inhalation with 1–2% isoflurane and decapitated to obtain the cortical region. The tissue (a pool of cortices obtained from two animals) was dissected in HBSS (Hank’s balanced salt solution; Gibco, Grand Island, NY, USA, catalog number 14175079) in an adequately sterilized environment. Afterwards, the cortex was enzymatically dissociated with trypsin (0.05%; Gibco, catalog number 15090046) at 37 °C for 15 min, and mechanical dissociation was then performed with a fine-tuned Pasteur pipette for 15 min. The homogenate obtained was centrifuged at 1400 rpm for 5 min, before performing the second enzymatic dissociation in HBSS containing 40 U of papain/mL (Merck KGaA, Darmstadt, Germany, catalog number 1.07144.0025), 0.02% cysteine (Sigma-Aldrich, St. Louis, MO, USA, catalog number 168149) and 0.003% DNAse (Sigma-Aldrich, catalog number D5025), with mechanical dissociation for 15 min and centrifugation at 1400 rpm for 5 min. After resuspension of the pellet in HBSS, the suspension was left to decant for 30 min. The supernatant was then collected and centrifuged at 1400 rpm for 7 min. The cell pellet obtained was resuspended in DMEM/F12 (Dulbecco’s Modified Eagle Medium/Nutrient Mixture F12; Gibco, catalog number 12500062) containing 10% fetal bovine serum (FBS; Gibco, catalog number 12657029), HEPES (15 mM; Sigma-Aldrich, catalog number H3784), NaHCO_3_ (14.3 mM), amphotericin B (2.5 μg/mL; Gibco, catalog number 15290026) and gentamicin (0.05 mg/mL; Gibco, catalog number 15710072), and the cells were seeded in 6- or 24-well plates at a density of 2–4 × 10^5^ cells/cm^2^, on plates previously coated with poly-L-lysine. The cells were cultured in an incubator at a temperature of 37 °C with 5% CO_2_, with periodic changes (every 3 days) of culture medium. From the third week of culture, the astrocytes received DMEM/F12 supplemented with 20% FBS, being maintained until confluence, reached at approximately 30 days in vitro.

The culture of cortical astrocytes from neonatal animals (1–3 days of age) was performed as previously described [20]. The cortex was removed and dissected, removing the meninges. Subsequently, the tissue was suspended in HBSS and enzymatically dissociated with trypsin (0.05%) and DNAse (0.003%) at 37 °C for 7 min, followed by mechanical dissociation with a fine-tuned glass Pasteur for 10 min. The contents were centrifuged at 1000 rpm for 5 min, with subsequent resuspension of the pellet in HBSS and mechanical dissociation. The supernatant was collected after decantation and centrifuged for 7 min at 1000 rpm. The pellet was resuspended with DMEM/F12 culture medium supplemented with 10% FBS, HEPES (15 mM), NaHCO_3_ (14.3 mM) and supplemented with amphotericin B (2.5 μg/mL) and gentamicin (0.05 mg/mL). The cells were seeded in 24-well plates previously coated with poly-L-lysine at a density of 3–5 × 10^5^ cells/cm^2^. The culture was maintained for approximately 30 days, until reaching confluence, under the same conditions as those used for adult rodent culture. The cells were supplemented with 20% FBS from the third week of culture. As previously demonstrated by our group [14], this protocol yields cultures with >95% astrocyte purity. This characterization was based on immunostaining for the astrocytic marker glial fibrillary acidic protein (GFAP), with minimal microglial or neuronal contamination, confirmed by staining for CD11b/c (a specific microglial protein) and β-tubulin III and NeuN (neuronal microtubule and nuclei proteins, respectively).

**Figure 1:**
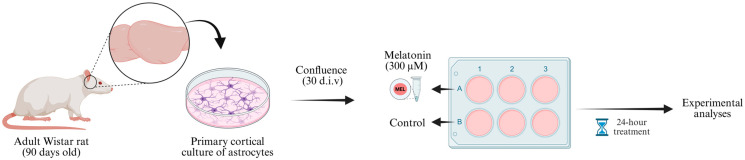
Experimental design. Cortices from 90-day-old male Wistar rats were dissected (two animals pooled per culture) and processed to obtain primary astrocyte cultures. The cells were maintained in DMEM/F12 medium (supplemented with 10% FBS during the first two weeks and 20% FBS from the third week onward) until reaching confluence (approximately 30 d.i.v.). Then, astrocyte cultures were divided into two experimental groups: control (DMEM/F12 + 1% FBS) and melatonin (solubilized in DMEM/F12 + 1% FBS). After 24 h incubation, samples were collected for subsequent molecular and biochemical analyses. d.i.v., days in vitro. Created in BioRender. Bobermin, L. (2025) https://BioRender.com/6xrqy8a (accessed on 29 November 2025).

After cell confluence, the wells of the astrocyte culture plates were divided into two treatment groups: basal (control condition) and melatonin (Figure 1). For the basal condition, on the day of treatment, the culture medium was replaced with DMEM/F12 with 1% FBS. In parallel, for the melatonin-treated group, the cell culture medium was replaced with DMEM/F12 with 1% FBS containing melatonin at a final concentration of 300 µM. Both groups were maintained for 24 h in the incubator under the standard conditions described.

### 2.3. Cell Morphology and Viability

The morphology of astrocyte cultures was observed under a phase contrast microscope, and images were captured with a mounted camera (Nikon microscope with DXM1200C digital camera, Tokyo, Japan). To assess cell viability, the tetrazolium salt reduction (MTT; Sigma-Aldrich, catalog number M5655) to formazan method was used. The assay consists of adding MTT (final concentration of 50 µg/mL) to the culture medium with incubation for 3 h [21]. Afterwards, the medium is removed, and 0.3 mL of dimethyl sulfoxide (DMSO) is added with stirring for 5 min to solubilize the formazan crystals produced [22]. The resulting absorbance was measured at 560 and 650 nm and the results were expressed as a percentage of the control conditions. 

### 2.4. Lactate Levels

Lactate levels were quantified using a commercial kit (Bioclin, Belo Horizonte, MG, Brazil, catalog number K084). Lactate dehydrogenase (LDH) catalyzes the oxidation of L-lactate to pyruvate, with the consequent reduction of NAD^+^ to NADH. The concentration of L-lactate is measured by the increase in absorbance at 340 nm resulting from NADH formation. For this purpose, 100 µL of extracellular medium collected 24 h after melatonin treatment was used for the experiment. The samples were analyzed with a spectrophotometric microplate reader at an absorbance of 340 nm. The lactate concentrations (mM) in the samples were calculated based on the absorbance of the standard provided in the kit.

### 2.5. Glutamine Synthase Activity (GS)

The protocol was performed as previously described [23]. Briefly, 0.1 mL of the reaction mixture with the following components was added: 10 mM MgCl_2_, 50 mM L-glutamate, 100 mM imidazole-HCl buffer (pH 7.4), 10 mM 2-mercaptoethanol, 50 mM hydroxylamine-HCl and 10 mM ATP to the cell homogenate (0.1 mL) with subsequent incubation for 15 min at 37 °C. The reaction was stopped by adding 0.4 mL of a solution containing: 370 mM ferric chloride, 670 mM HCl and 200 mM trichloroacetic acid. After centrifugation, the absorbance was measured in the supernatant of the samples at 530 nm and compared with the absorbances resulting from the standard curve prepared with γ-glutamylhydroxamate (Sigma-Aldrich, catalog number G2253) treated with a ferric chloride reagent. The results were calculated and expressed in µmol/mg protein/h.

### 2.6. RNA Extraction and Quantitative RT-PCR

After melatonin treatment for 24 h, the RNA was extracted using TRIzol reagent (Sigma-Aldrich, catalog number T9424). The concentration and purity of the extracted RNA were measured using spectrophotometry and a 260:280 ratio. To obtain complementary DNA (cDNA), 1 µg of total RNA was used and reverse transcribed using the High-Capacity Reverse Transcription kit (Applied Biosystems/Thermo Fisher, Waltham, MA, USA catalog number 4368814), following the manufacturer’s instruction. The messenger RNA (mRNA) encoding *β-actin* (#Rn00667869_m1), *GS* (Rn01483107_m1), *GCL* (Rn00689046_ m1), *NFκB p65* (Rn01502266_m1), *COX2* (Rn01483828_m1), *iNOS* (#Rn00561646_m1), *NLRP3* (#Rn04244620_m1), *SOD1* (#Rn00566938_m1), *SOD2* (#Rn00690588_g1), *Nrf2* (Rn00582415_m1), *HO-1* (Rn01536933_ m1), *AMPK* (Rn00576935_m1), *PGC-1α* (Rn00580241_m1) and *SIRT1* (Rn01428096_m1) was analyzed using the QuantStudio 1 real-time PCR system and the TaqMan probes listed above, purchased from Applied Biosystems. To normalize the target mRNA levels, *β-actin* was used. The results were analyzed using the 2^−ΔΔCt^ method and expressed in relation to the levels of the control group [24].

### 2.7. Western Blotting

The protocol was performed as previously described [25]. The samples were prepared with a solution containing 4% SDS, and the proteins were denatured for 5 min at a temperature of ≥95 °C. Subsequently, the samples were centrifuged at 10,000× *g* for 5 min and the supernatant was aliquoted, of which 5 µL were used for protein dosage. The samples (10 μg of protein) were subjected to SDS-polyacrylamide gel electrophoresis and fixed on nitrocellulose membranes with 0.45 µm pores. The transfer was confirmed using Ponceau S and the membranes were exposed to a 2% bovine serum albumin solution in T-TBS for 2 h at 37 °C. The membranes were incubated overnight (4 °C) with anti-GFAP (1:1000; Sigma-Aldrich, catalog number G9269) or anti-PGC-1α (1:500; Cell Signaling, Danvers, MA, USA, catalog number 2178). Subsequently, the membranes were incubated with the secondary antibody (IgG) coupled to peroxidase (dilution 1:10,000, Elabscience, Wuhan, China, catalog E-AB-1058) anti-mouse or anti-rabbit for 2 h at 37 °C under shaking. After detection, the membranes were reused after exposure to hydrogen peroxide to remove previous signals, as well as with successive washes with T-TBS at each stage. For signal detection, the ECL Super Signal West Pico Plus (Thermo Fisher, Waltham, MA, USA, catalog number 34580) was used and detected by Chemidoc MP (Bio-Rad Laboratories, Hercules, CA, USA). The analysis was performed using ImageJ software (1.54g, National Institutes of Health, USA) with data normalization by β-actin content (mouse anti-β-actin antibody, Santa Cruz, CA, USA, catalog number sc-4778). Complete immunoblots corresponding to all quantitative data from this study are compiled in Supplementary Figure S1.

### 2.8. Determination of Reactive Species Production

As described in previous studies, the determination of reactive species was performed using the DCFH oxidation method [26]. The 2′,7′-dichlorofluorescein diacetate (DCFH-DA) reagent (Sigma-Aldrich, catalog number D6883) was added to the cells at a final concentration of 10 µM with subsequent incubation for 30 min at 37 °C. Afterwards, the cells were lysed in 0.3 mL PBS/Triton (0.2%) and centrifuged, and the fluorescence was measured in the supernatant at excitation and emission wavelengths of 485 nm of 520 nm, respectively. The results were expressed as percentages relative to the control condition.

### 2.9. Reduced Glutathione (GSH) Content

As previously described, after the treatment period, the cells were homogenized in 100 mM sodium phosphate buffer (pH 8.0) containing 5 mM EDTA, in which the protein was precipitated with 1.7% metaphosphoric acid [27]. The supernatant was incubated with ophthaldialdehyde (1 mg/mL in methanol) for 15 min at room temperature. Finally, fluorescence was determined at 350 and 420 nm of excitation and emission, respectively. The calibration curve was performed with standard GSH solution (0–500 μM; Sigma-Aldrich, catalog number G4251).

### 2.10. Measurement of Cytokine Levels

To evaluate extracellular concentrations of TNF-α (Invitrogen, Carlsbad, CA, USA, catalog number 88–7340-22), IL-1β (Invitrogen, catalog number BMS630), IL-6 (Invitrogen, catalog number BMS625), and IL-10 (Invitrogen, catalog number BMS629), the ELISA (Enzyme-Linked Immunosorbent Assay) methodology was used employing commercial kits. The procedures were performed according to the manufacturer’s instructions and previous work by the group [28]; results are expressed in pg/mL.

### 2.11. Protein Quantification

Protein quantification for the methodologies described in this study was performed according to the Lowry method, and bovine serum albumin was used as a standard for quantification [29].

### 2.12. Statistical Analysis

Data are expressed as mean ± standard deviation (SD) from independent samples. Normality was assessed using the Shapiro–Wilk test. For comparisons between two independent groups (control vs. melatonin), statistical differences were evaluated using Student’s *t*-test for independent samples, as the data were normally distributed. For the concentration-response involving multiple melatonin doses, one-way ANOVA followed by Tukey’s post hoc test was applied. *p*-values < 0.05 were considered significant. *p*-values, t-values, degrees of freedom (df), difference between means, 95% confidence interval (CI) range, and the standardized effect size (Cohen’s d) are detailed in Supplementary Table S1.

## 3. Results

### 3.1. Melatonin Does Not Alter Astrocyte Viability, Morphology, or Metabolic Parameters

**Figure 2:**
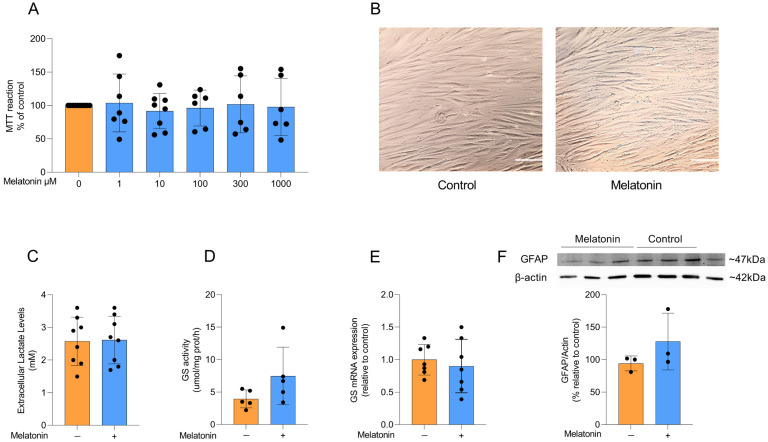
Effects of melatonin on adult cortical astrocyte viability, morphology, lactate and astrocytic markers. (**A**) Effects of melatonin on cell viability in adult rodent astrocyte cultures at doses of 1, 10, 100, 300, and 1000 µM. (**B**) Representative images of astrocyte cultures under control conditions and after treatment with 300 µM melatonin. Scale bar = 40 µm (magnification of 200×). Effects of melatonin treatment (300 µM) on (**C**) extracellular lactate levels, (**D**) GS activity and (**E**) mRNA expression, and the protein content of (**F**) GFAP. Orange bars represent control group, and blue bars represent melatonin-treated group. Data are expressed as mean ± SD (n = 9 independent biological replicates for MTT; n = 8 independent biological replicates for lactate; n = 5 independent biological replicates for GS activity; n = 7 independent biological replicates for *GS* mRNA; n = 3 independent biological replicates for GFAP immunocontent). Each biological replicate represents an independent culture (with each culture derived from a pooled preparation of cortices from two animals). Cell viability across multiple concentrations was analyzed using one-way ANOVA followed by Tukey’s post hoc test (**A**). All two-group comparisons (control vs. melatonin) were analyzed using Student’s *t*-test for independent samples. *p* < 0.05 were considered significant (*p*-values were described for each parameter in the Results section).

Initially, we assessed the viability of adult astrocyte cultures exposed to increasing concentrations of melatonin (1, 10, 100, 300, and 1000 µM) for 24 h. Melatonin did not affect MTT reduction at any concentration tested (F _(5, 37)_ = 0.1364, *p* = 0.9828), indicating that cell viability remained unchanged relative to control conditions (Figure 2A). Therefore, based on these data, on our group’s previous study with melatonin, and on the reported variability of melatonin concentrations (0.1 to 1000 µM) in several in vitro studies, we chose the concentration of 300 µM melatonin for the next experiments [30,31,32,33,34]. In addition, this choice was based on a treatment that exceeded the physiological concentrations of melatonin to investigate its potential pharmacological actions, as well as to promote specific cellular responses. The treatment time (24 h) with melatonin was based on those widely described in the literature to allow the comparison of the results obtained in this study with those in the literature [30,31].

Astrocytes in culture exhibit a typical polygonal and fusiform morphology, and melatonin treatment did not induce any morphological changes compared to control conditions (Figure 2B). Lactate production and conversion of glutamate into glutamine through glutamine synthetase (GS) activity are physiological parameters of astrocytic function. To assess whether melatonin modulates these astrocyte parameters, we measured extracellular lactate levels after treatment with 300 µM melatonin for 24 h and observed no significant difference compared to control conditions (Figure 2C; t = 0.1022, df = 14, *p* = 0.9201). We further examined the activity and expression of GS (Figure 2D and E, respectively), a key astroglial enzyme involved in the glutamate–glutamine cycle [11], but found that melatonin did not change either parameter under basal conditions (t = 1.706, df = 8, *p* = 0.1264 for GS activity; t = 0.5433, df = 12, *p* = 0.5969 for *GS* expression). Consistently, the content of the classical astrocyte marker GFAP (Figure 2F) was also not modified by melatonin treatment (t = 1.293, df = 4, *p* = 0.2657).

### 3.2. Effects of Melatonin on Redox Homeostasis 

**Figure 3:**
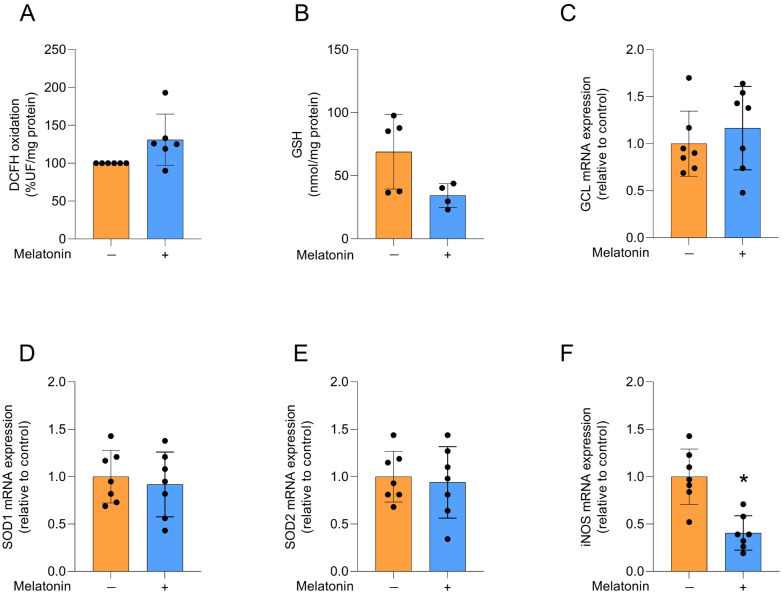
Redox status and related transcripts in adult cortical astrocytes treated with melatonin (300 µM, 24 h). Redox status: (**A**) DCFH oxidation (indicative of reactive species levels) and (**B**) GSH content. Expression of genes encoding: (**C**) *GCL*, (**D**) *SOD1*, (**E**) *SOD2* and (**F**) *iNOS*. Orange bars represent control group, and blue bars represent melatonin-treated group. Data are expressed as mean ± SD (n = 6 independent biological replicates for DCFH oxidation; n = 4 independent biological replicates for GSH levels; n = 7 independent biological replicates for gene expression evaluations). Each biological replicate represents an independent culture (with each culture derived from a pooled preparation of cortices from two animals). Statistical differences between control and melatonin groups were analyzed using Student’s *t*-test for independent samples. *p* < 0.05 was considered statistically significant (*p*-values were described for each parameter in the Results section). * Indicates difference in relation to the control condition.

We evaluated redox homeostasis by first assessing the production of reactive oxygen species (ROS) through DCFH oxidation (Figure 3A), which was not significantly affected by melatonin treatment (t = 2.242, df = 10, *p* = 0.068). We then examined the content of the major non-enzymatic antioxidant GSH (Figure 3B; t = 2.235, df = 7, *p* = 0.0605) and the mRNA expression of *GCL*, the regulatory enzyme for its synthesis (Figure 3C; t = 0.7799, df = 12, *p* = 0.4506), and similarly observed no changes following melatonin exposure. The expression of the antioxidant enzyme isoforms *SOD1* and *SOD2* (Figure 3D,E, respectively; t = 0.4892, df = 12, *p* = 0.6335 for *SOD1*; t = 0.3512, df = 12, *p* = 0.7315), which act on superoxide anions, was also not affected by melatonin treatment. In contrast, the expression of *iNOS*, the enzyme responsible for producing nitric oxide, was decreased by melatonin (Figure 3F; t = 4.566, df = 12, *p* = 0.0006).

### 3.3. Effects of Melatonin on the Inflammatory Profile

**Figure 4:**
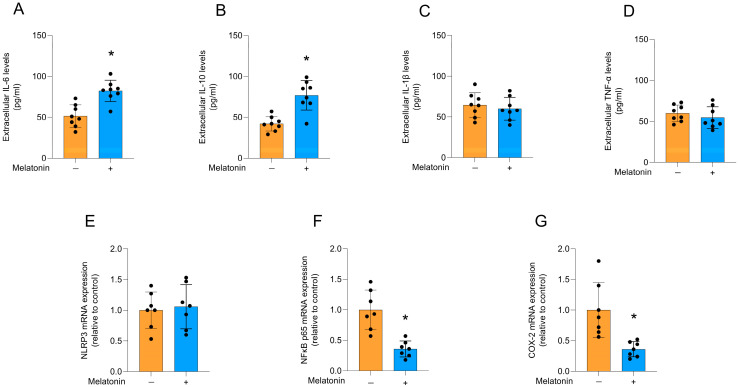
Cytokine release and inflammatory pathways in adult cortical astrocytes after melatonin incubation (300 µM, 24 h). Extracellular cytokines: (**A**) IL-6, (**B**) IL-10, (**C**) IL-1β, (**D**) TNF-α. Expression of genes encoding: (**E**) *NLRP3*, (**F**) *NFκB p65*, and (**G**) *COX-2*. Orange bars represent control group, and blue bars represent melatonin-treated group. Data are expressed as mean ± SD (n = 8 independent biological replicates for ELISA assays; n = 7 independent biological replicates for gene expression evaluations). Each biological replicate represents an independent culture (with each culture derived from a pooled preparation of cortices from two animals). Statistical differences between control and melatonin groups were analyzed using Student’s *t*-test for independent samples. *p* < 0.05 was considered statistically significant (*p*-values were described for each parameter in the Results section). * Indicates difference in relation to the control condition.

Melatonin increased the extracellular levels of IL-6 (Figure 4A; t = 4.574, df = 14, *p* = 0.0004) and IL-10 (Figure 4B; t = 4.865, df = 14, *p* = 0.0003) in cultured astrocytes, compared to the control condition. However, no significant differences were observed for the levels of IL-1β (Figure 4C; t = 0.6175, df = 14, *p* = 0.5468) and TNF-α (Figure 4D; t = 0.8955, df = 14, *p* = 0.3857), as well as the mRNA expression of the *NRLP3* inflammasome component (Figure 4E; t = 0.3240, df = 12, *p* = 0.7515). In addition, melatonin significantly decreased the mRNA expression of the key regulator of the inflammatory response, *NFκB* (Figure 4F; t = 4.825, df = 12, *p* = 0.0004), and its transcriptional target, the inflammatory enzyme, *COX-2* (Figure 4G; t = 3.626, df = 12, *p* = 0.0035), compared to the control conditions.

### 3.4. Potential Signaling Pathways Associated with the Effects of Melatonin

**Figure 5:**
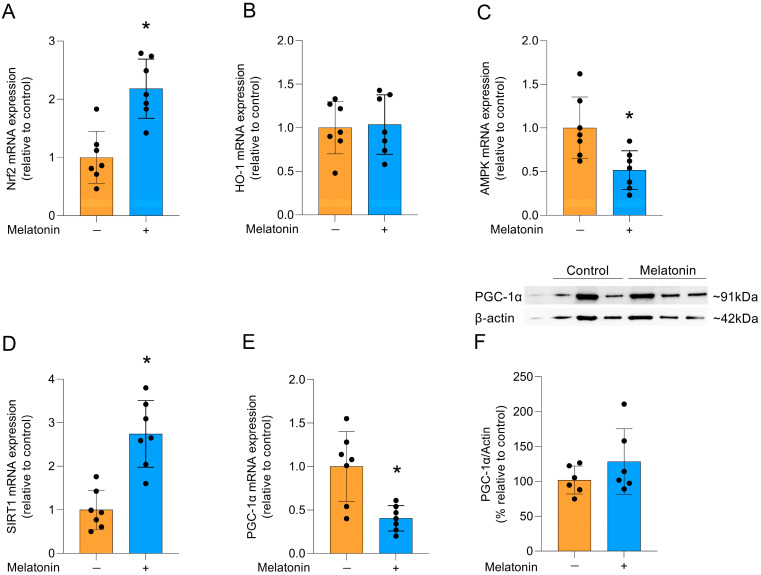
Signaling pathways linked to cytoprotection and PGC-1α protein in adult cortical astrocytes after melatonin incubation (300 µM, 24 h). Expression of genes encoding: (**A**) *Nrf2*, (**B**) *HO-1*, (**C**) *AMPK*, (**D**) *SIRT1* (**E**) *PGC-1α*. Protein content: (**F**) PGC-1α. Orange bars represent control group, and blue bars represent melatonin-treated group. Data are expressed as mean ± SD (n = 7 independent biological replicates for gene expression evaluations; n = 6 independent biological replicates for Western blotting analysis). Each biological replicate represents an independent culture (with each culture derived from a pooled preparation of cortices from two animals). Statistical differences between control and melatonin groups were analyzed using Student’s *t*-test for independent samples. *p* < 0.05 was considered statistically significant (*p*-values were described for each parameter in the Results section). * Indicates difference in relation to the control condition.

Melatonin can modulate several signaling pathways, including that of the Nrf2 transcription factor, which activates cytoprotective genes [35]. Accordingly, melatonin incubation increased the mRNA expression of *Nrf2* (Figure 5A; t = 4.628, df = 12, *p* = 0.0006). However, there was no difference in the expression of its transcriptional target, the HO-1 enzyme (Figure 5B; t = 0.2168, df = 12, *p* = 0.8320). Additionally, the gene expression of *AMPK* (Figure 5C; t = 3.069, df = 12, *p* = 0.0097) was significantly reduced and melatonin significantly increased the mRNA expression of *SIRT1* (Figure 5D; t = 5.178, df = 12, *p* = 0.0002), another pathway associated with cytoprotection. In contrast, the gene expression of *PGC-1α* was significantly reduced (Figure 5E, t = 3.668, df = 12, *p* = 0.0032), but there was no significant change in the quantification of PGC-1α protein levels (Figure 5F; t = 1.274, df = 10, *p*= 0.2316).

### 3.5. Effects of Melatonin on Cell Viability, Lactate Levels, Astroglial Parameters and Redox Homeostasis in Cortical Astrocyte Cultures from Neonatal Animals

**Figure 6:**
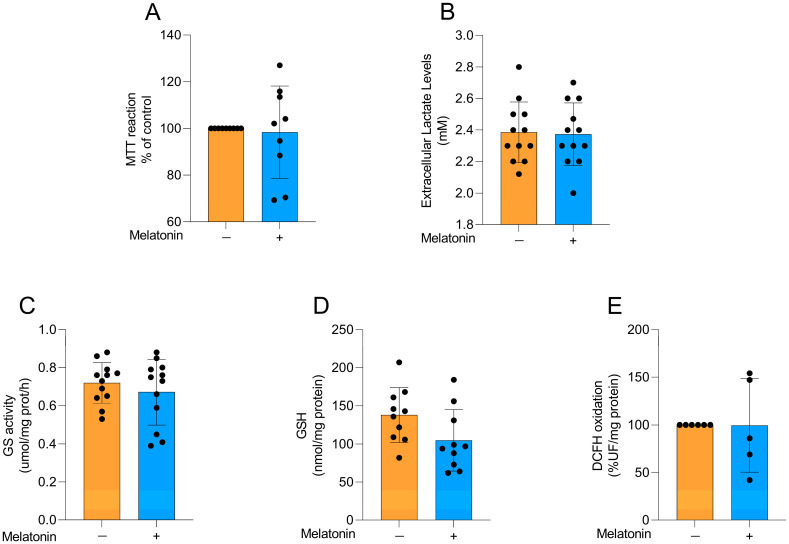
Basal metabolic and redox parameters in neonatal cortical astrocytes after melatonin incubation (300 µM, 24 h). (**A**) Cell viability, (**B**) extracellular lactate, (**C**) GS activity, (**D**) GSH content, and (**E**) DCFH oxidation (indicative of reactive species levels). Orange bars represent control group, and blue bars represent melatonin-treated group. Data are expressed as mean ± SD (n = 9 independent biological replicates for MTT; n = 12 independent biological replicates for lactate levels; n = 12 independent biological replicates for GS activity; n = 10 independent biological replicates for GSH levels; n = 5 independent biological replicates for DCFH oxidation). Each biological replicate represents an independent culture (with each culture derived from a pooled preparation of cortices from two animals). Statistical differences between control and melatonin groups were analyzed using Student’s *t*-test for independent samples. *p* < 0.05 was considered statistically significant (*p*-values were described for each parameter in the Results section).

To explore whether the effects of melatonin on astrocytes were age-dependent, we performed some experiments in astrocyte cultures obtained from newborn Wistar rats, particularly exploring metabolic and redox parameters at the same concentration chosen for adult astrocytes. Cellular viability (Figure 6A; t = 0.2455, df = 16, *p* = 0.8092), extracellular lactate levels (Figure 6B; t = 0.1566, df = 22, *p* = 0.8770), GS activity (Figure 6C; t = 0.8104, df = 22, *p*= 0.4264), GSH content (Figure 6D; t = 1.949, df = 18, *p* = 0.0671), and reactive species production (Figure 6E, t = 0.02018, df = 9, *p* = 0.9843) were evaluated in the cells. No changes were observed in these parameters following the incubation of the cells with 300 µM melatonin for 24 h, similarly to our findings in adult astrocytes.

## 4. Discussion

Recent clinical studies have examined the neuroprotective potential of melatonin in different neurological conditions. In individuals with dementia, melatonin has been explored for its potential to reduce amyloid burden [36]. In Parkinson’s disease, randomized trials suggest beneficial effects on sleep quality and possibly motor symptoms [37]. Moreover, a recent meta-analysis also indicates improvements in cognition in adults with cognitive impairment [38]. Considering the therapeutic potential of melatonin, it is important to explore the underlying cellular and molecular mechanisms in the CNS. Our results explored the effects of melatonin on classical astrocytic functions, such as inflammatory response, redox homeostasis, and related signaling pathways, in cortical astrocyte cultures obtained from adult rodents. Melatonin modulated some glial responses in mature astrocytes, demonstrating glioprotective properties. It is important to note that adult astrocytes present well-established connections and are more organized than those from newborn tissue, which are more plastic. Although melatonin is classically described as a potent antioxidant molecule, the concentration used in our study was not capable of modulating reactive species production or enhancing antioxidant defenses in astrocytes, in either neonatal or adult cell cultures [39,40]. On the other hand, melatonin modulated crucial signaling pathways for neuroprotection/glioprotection. The lack of change in several parameters under basal conditions likely reflects the absence of a redox challenge rather than insufficient melatonin action. Future studies incorporating stressors such as LPS or H_2_O_2_ will be important to determine whether the effects of melatonin on adult astrocytes extend to conditions of oxidative or inflammatory challenge.

Lactate is essential as an energy substrate for the brain in addition to its roles in memory and learning, neuronal plasticity and synaptic activity [41,42,43]. Moreover, the activity of the GS enzyme is crucial for glutamate recycling in astrocytes to maintain CNS homeostasis [44]. In parallel with metabolic functions, GFAP is a classic astrocytic cytoskeletal marker that reflects structural and functional adaptations of astrocytes, with its expression dynamically changing during neurodevelopment and aging, often in association with vimentin [45,46]. Although several studies have shown that melatonin can regulate most of these parameters in vivo and in vitro, we did not observe any difference in cultured astrocytes from adult and newborn animals [47,48,49,50,51,52]. As previously mentioned, this lack of change may be due to the absence of stressful stimuli, as well as the specific concentration and incubation time used. Supporting this context-dependent action, melatonin has been shown to reduce astrocyte reactivity following brain injury, whereas in our unchallenged cultures, it did not modify GFAP or vimentin [52].

The high energy demand of the brain makes it particularly vulnerable to ROS-induced homeostatic imbalance, a process exacerbated with age [53,54]. GSH is often highlighted as a target for melatonin in studies, reinforcing its protective potential as a non-enzymatic antioxidant [34,55]. As such, we evaluated the expression of the enzyme that limits GSH synthesis, GCL, and the content of this potent antioxidant molecule after treatment with melatonin. No significant difference was observed, consistent with evidence indicating significant changes in this antioxidant after melatonin treatment in the presence of cellular redox stress [56]. To further characterize astroglial antioxidant defenses, the expression of SOD1 and SOD2, which are enzymes that regulate ROS and are controlled by the transcription factors Nrf2 and NFκB, was also examined [57]. SOD1, SOD2 and DCFH oxidation analyses confirmed the absence of significant differences in ROS levels and in the expression of these enzymes, suggesting a controlled cellular environment without external challenges. Such conditions may be insufficient to activate melatonin’s antioxidant effects via SOD, which are typically reported under situations of challenge [58,59]. According to previous studies, melatonin MT_1_/MT_2_ receptors reach saturation at concentrations lower than those used in this study (300 µM), implying that antioxidant effects of melatonin at high doses may represent receptor-independent mechanisms and appear to be context-dependent, without impacting the basal redox state of healthy [60]. However, melatonin decreased the mRNA expression of *iNOS* in cultured astrocytes, consistent with previous studies investigating melatonin’s role in iNOS suppression and its glioprotective effects [61,62]. Interestingly, this effect may be associated with the immunomodulatory role of the iNOS enzyme, mediated by the release of pro-inflammatory cytokines [63].

With regard to the astrocytic inflammatory response, melatonin increased extracellular levels of both IL-6 and IL-10 but did not change IL-1β release. It is evident from the literature that both IL-6 and IL-10 exhibit different actions in inflammatory or anti-inflammatory contexts, depending on the cellular environment [64,65,66]. Thus, it was of interest to analyze the effect of TNF-α cytokine, which can control the activity of IL-1β, via NLRP3, an inflammasome component involved in IL-1β processing [67,68]. However, no significant difference was observed in TNF-α levels after melatonin treatment, at the concentration tested. Therefore, potential anti-inflammatory and homeostatic roles of IL-6 and IL-10 can be hypothesized. The concentration of 300 µM melatonin exceeds typical physiological ranges [69,70], but it is important to note that we performed a cellular viability curve up to 1 mM melatonin, and none of the tested concentrations reduced cell viability. Notably, melatonin has been shown dose-dependent anti-inflammatory effects in challenged models, NLRP3 suppression typically requires prior activation, explaining its unaltered state in our basal conditions [71,72,73,74]. In line with the inflammatory profile, NFκB is a key transcription factor in the inflammatory response. Previous studies have reported an increase in NFκB expression in cortical tissue with aging; however, melatonin treatment has been shown to reverse this process in young and old animals [75,76]. Similarly, our study demonstrated a reduction in the expression of this key inflammatory factor, along with COX-2, an enzyme involved in prostaglandin production. Previous studies have shown that ramelteon, a melatonin receptor agonist, was able to reduce the expression of *NFκB* and *COX-2* in neonatal rodent cultures [77]. In agreement with these findings, we demonstrated that adult rodent cultures exhibit similar effects on these parameters following melatonin treatment [77].

Previous studies by our group have demonstrated the key role of Nrf2 in glioprotection, since this transcription factor modulates antioxidant and anti-inflammatory responses [18,20,28,78]. Melatonin displays synergistic effects with resveratrol by increasing the expression of *Nrf2* and *HO-1*, but its effects when administered alone do not include HO-1 [31]. Our findings align with previous literature, in which melatonin increased the expression of Nrf2, but not HO-1, suggesting that this transcriptional factor is critical for the glioprotective effects of melatonin in mature astrocytes. The activation of Nrf2 without concomitant induction of HO-1 may be attributed to specific characteristics of the cortical tissue [79]. Therefore, the protective actions of melatonin may derive from Nrf2 activation leading to NFκB suppression [31], independently of HO-1 induction. Moreover, it can be hypothesized that a more prolonged exposure to melatonin may allow the upregulation of *Nrf2* to be translated into the activation of its downstream target genes. This response also suggests that melatonin may act as a preparatory stimulus by activating important pathways that increase cellular resilience to subsequent challenges, without necessarily altering basal redox parameters [30].

**Figure 7:**
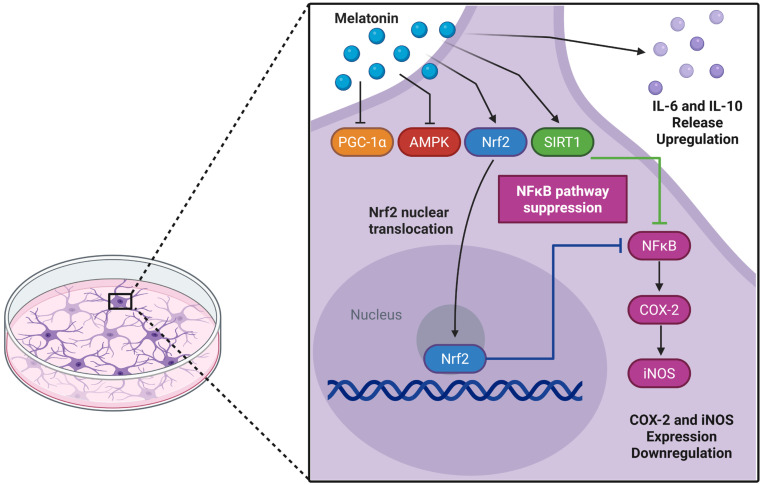
Potential glioprotective mechanisms of melatonin in cultured cortical astrocytes from adult Wistar rats. Melatonin may trigger a coordinated cytoprotective response through activation of the Nrf2 and SIRT1 pathways. Nuclear translocation of Nrf2 can subsequently suppress the NFκB pathway, reducing the expression of pro-inflammatory enzymes COX-2 and iNOS, while increasing IL-6 and IL-10 levels. Upregulation of SIRT1 expression may further reinforce the anti-inflammatory and antioxidant responses. In addition, the transcriptional profile induced by melatonin suggests a regulatory mechanism that prevents excessive cellular activation and maintains astroglial homeostasis. Created in BioRender. Bobermin, L. (2025) https://BioRender.com/c5ez3rq (accessed on 29 November 2025).

AMPK plays a crucial role in brain energy regulation and is significantly affected by aging [80,81,82]. While previous studies identified melatonin as an AMPK activator in traumatic brain injury models [83], we observed *AMPK* downregulation in adult astrocyte cultures. In parallel, there was a reduced *PGC-1α* transcript, an important AMPK modulator, without a corresponding change in protein, suggesting a decoupling between transcription and protein abundance. This pattern is compatible with post-transcriptional buffering (e.g., altered translation efficiency or mRNA stability) and/or differences in protein turnover and post-translational control (acetylation/phosphorylation) that preserve protein levels despite lower mRNA [84,85,86,87,88]. In our experimental model, the effects of melatonin do not seem to involve AMPK/PGC-1α transcriptional activation; however, direct effects on the proteins in this axis cannot be ruled out. Of note, PGC-1α may be modulated post-translationally by SIRT1, which was upregulated by melatonin [3,89]. SIRT1-mediated deacetylation can stabilize PGC-1α and enhance its activity, while also contributing to melatonin-mediated protection against neuroinflammation and redox imbalance [3,90,91]. Although this study lacks a mechanistic approach to validate the involvement of specific signaling pathways, a limitation that should be addressed in future studies, the action of melatonin in adult astrocyte culture appears to involve the SIRT-1/Nrf2 pathway, in which SIRT1 may enhance PGC-1α activity and promote Nrf2 expression, ultimately contributing to cytoprotection (Figure 7).

Finally, in mature astrocyte cultures, melatonin treatment enhanced anti-inflammatory effects and activated key signaling pathways involved in glioprotection. The reduction in *AMPK-PGC-1α* pathway expression can be attributed to the increased expression of *SIRT1* and *Nrf2*, which effectively suppressed the *NFκB*, *iNOS*, and *COX-2*, attenuating the inflammatory response. Additionally, the lack of alteration in reactive species generation, *SOD1*, and *SOD2* further suggests that there were no redox challenges to mobilize AMPK and PGC-1α. Thus, this study contributes to the understanding of actions of melatonin in cultured mature astrocytes, a relatively unexplored area, and offers valuable insights into the effects of this versatile molecule following cellular insults.

## Data Availability

The datasets used and/or analyzed during the current study are available from the corresponding author on reasonable request, due to privacy reasons.

## Supplementary Materials

The following supporting information can be downloaded at: https://www.mdpi.com/article/10.3390/biomedicines13122967/s1, Table S1. Results of the statistical analysis; Figure S1. Western Blotting Membranes.
