# Supplementary material for: Melatonin Modulates Astrocyte Inflammatory Response and Nrf2/SIRT1 Signaling Pathways in Adult Rat Cortical Cultures
Source: Biomedicines. 2025 Dec 2;13(12):2967. doi: 10.3390/biomedicines13122967 (PMC12730976; doi:10.3390/biomedicines13122967)
Supplement: Supplementary file 1 [file biomedicines-13-02967-s001.zip › biomedicines-3966387-supplementary/Supplementary Materials/Supplementary Figure 1.pdf]

Supplementary Figure S1. Western Blotting Membranes

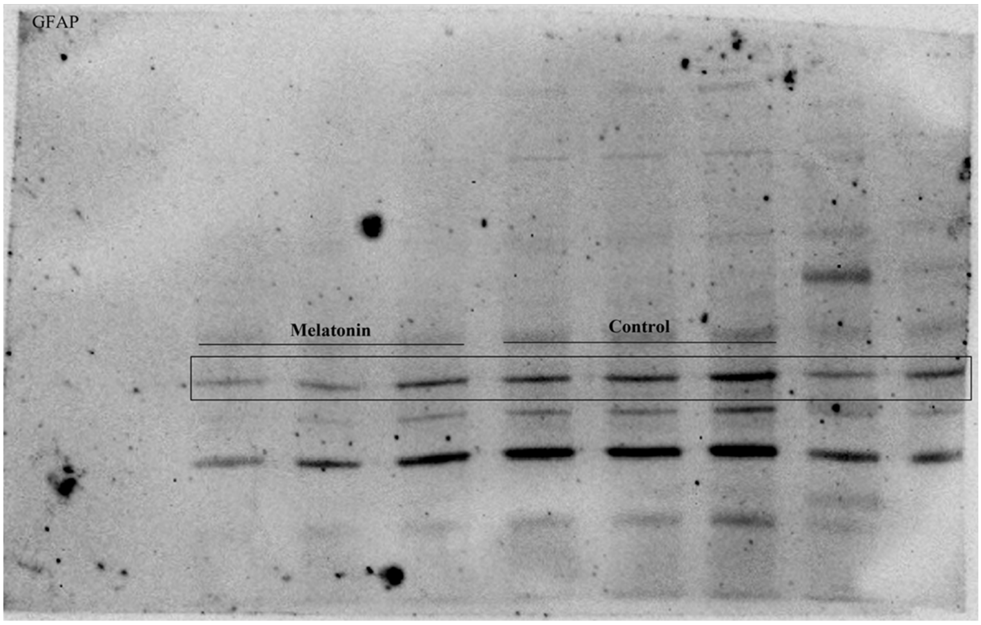

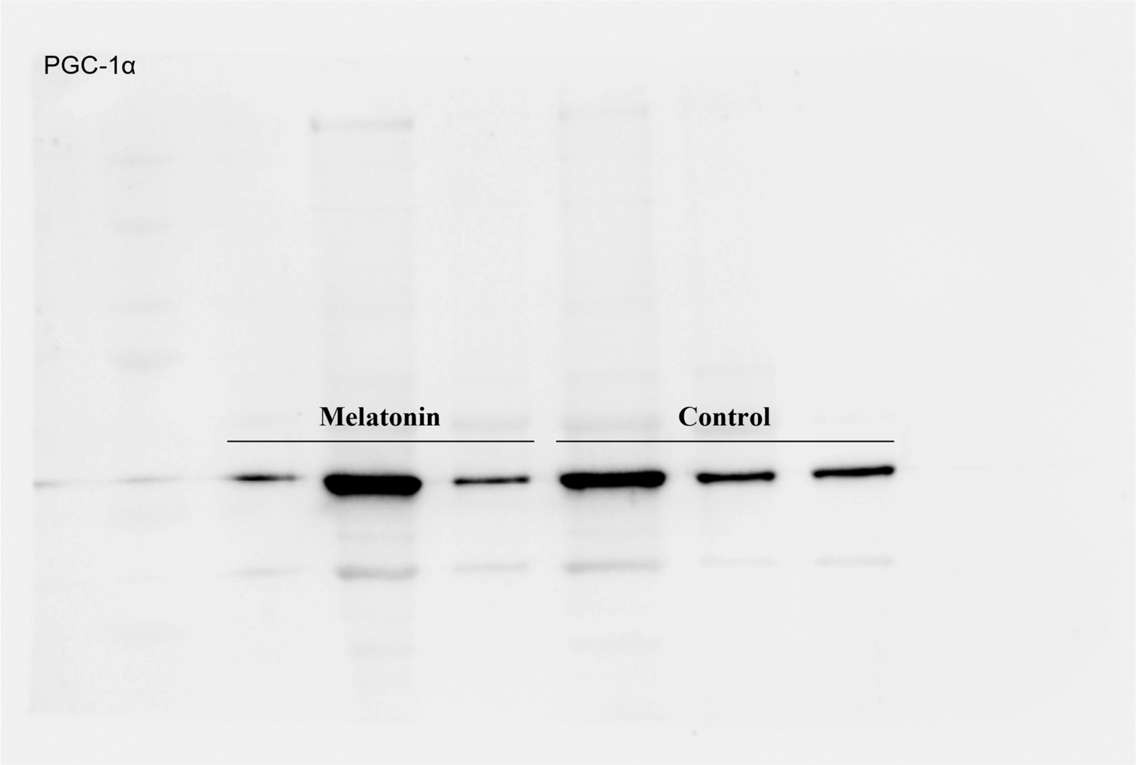
PGC-1α
Melatonin
Control

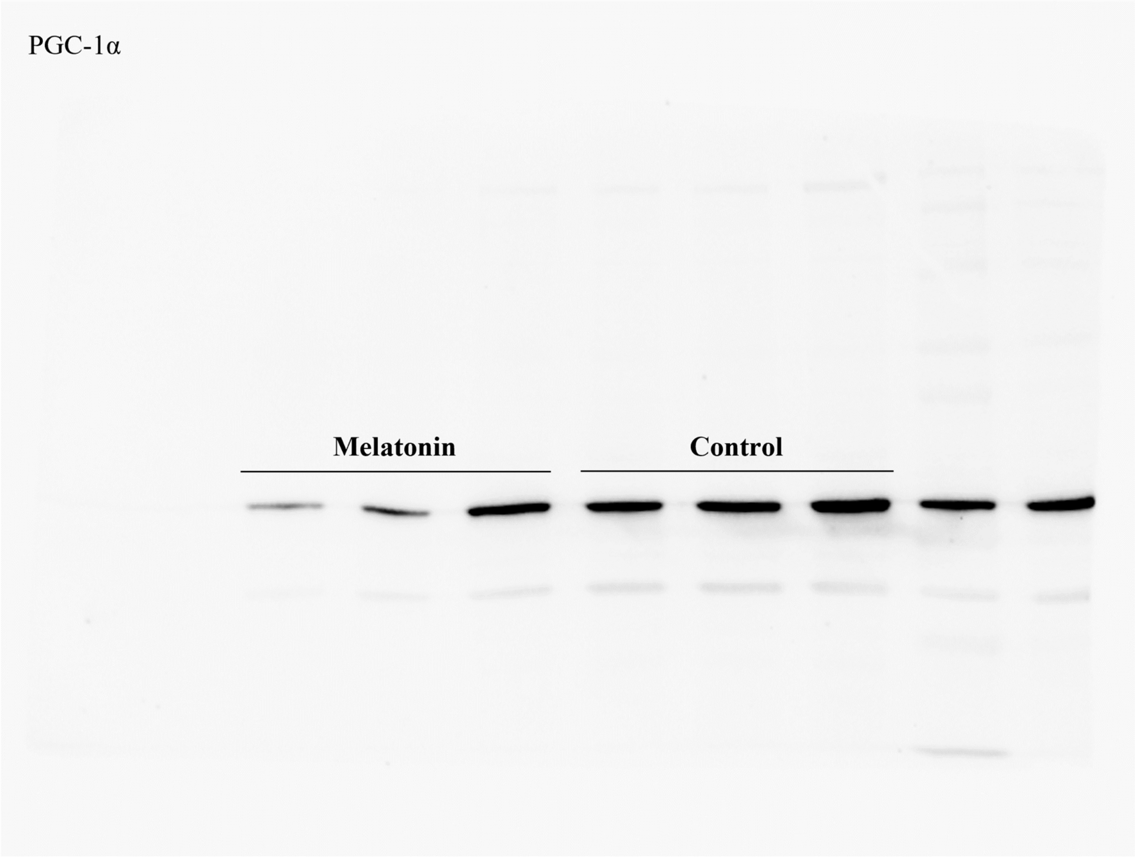
PGC-1α
Melatonin
Control

β-actin

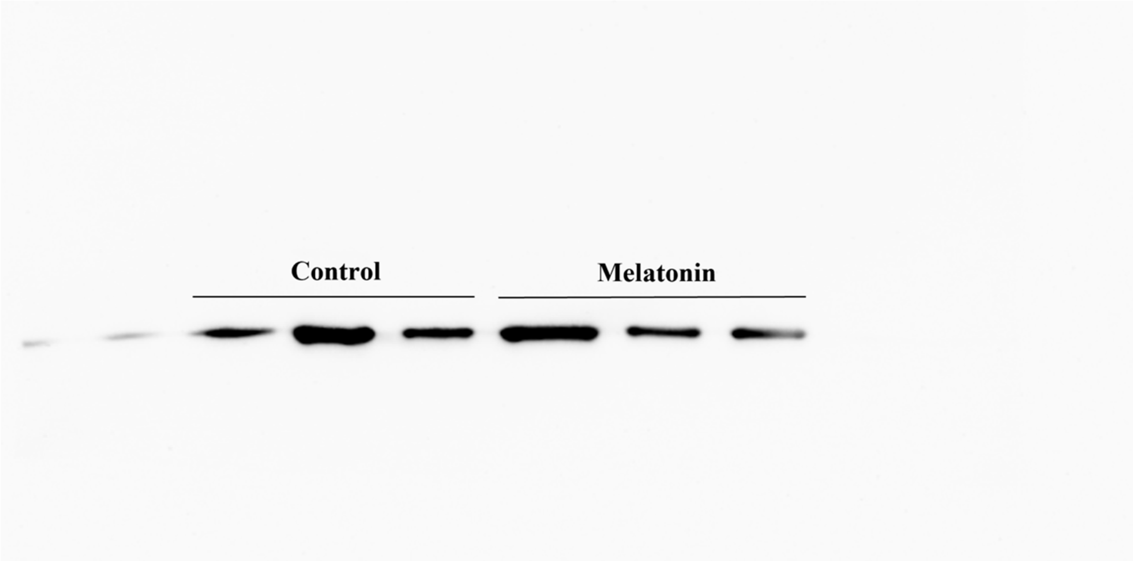

β-actin

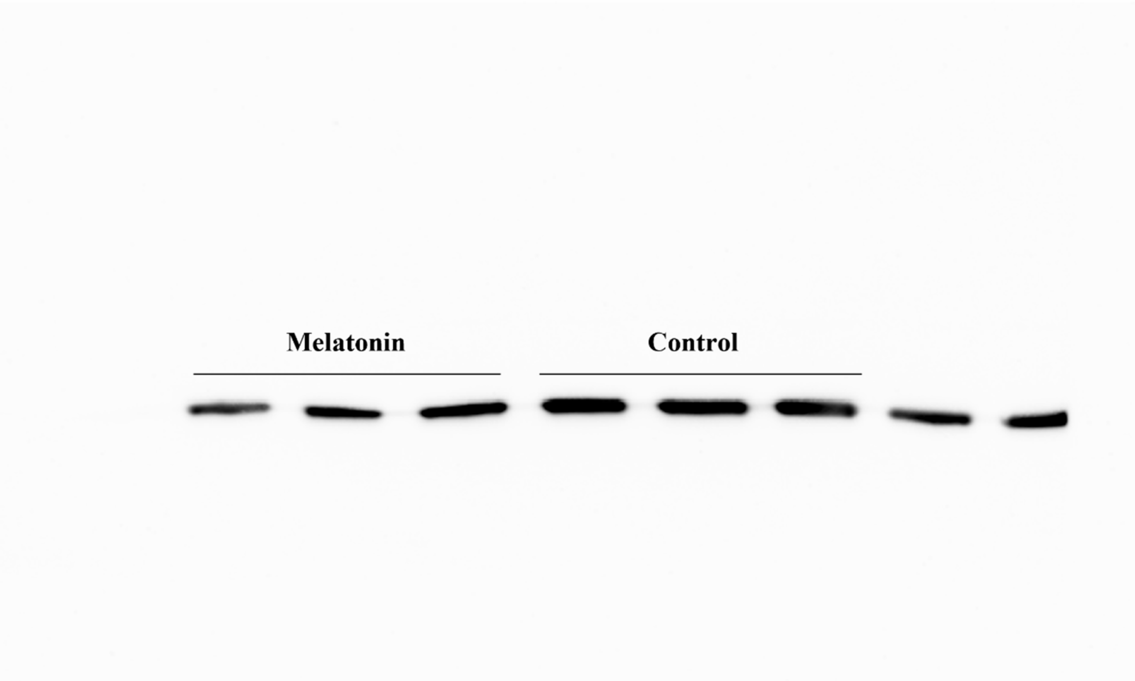

Membranes related to quantification of GFAP, PGC-1α and β-actin, respectively.
