# Supplementary material for: Melatonin Modulates Astrocyte Inflammatory Response and Nrf2/SIRT1 Signaling Pathways in Adult Rat Cortical Cultures
Source: Biomedicines. 2025 Dec 2;13(12):2967. doi: 10.3390/biomedicines13122967 (PMC12730976; doi:10.3390/biomedicines13122967)
Supplement: Supplementary file 1 [file biomedicines-13-02967-s001.zip › biomedicines-3966387-supplementary/Supplementary Materials/Supplementary Table 1.pdf]

**Supplementary Table S1.** Statistical summary of group comparisons

| Independent variable | n-value | t-value | Df | p-value | Difference between means | 95% CI | Effect size Cohen's d |
|---|---|---|---|---|---|---|---|
| Extracellular lactate levels | 8 | 0.1022 | 14 | 0.9201 | 0.03750 | -0.7497 to 0.8247 | 0.05 |
| GS activity | 5 | 1.7060 | 8 | 0.1264 | 3.540 | -1.245 to 8.325 | 1.08 |
| GS mRNA expression | 7 | 0.5433 | 12 | 0.5969 | -0.09714 | -0.4867 to 0.2924 | -0.29 |
| GFAP immunocontent | 3 | 1.2930 | 4 | 0.2657 | 33.70 | -38.68 to 106.1 | 1.05 |
| DCFH levels | 6 | 2.2420 | 10 | 0.068 | 31.00 | 0.1958 to 61.80 | 1.29 |
| GSH levels | 5 | 2.2350 | 7 | 0.0605 | -34.73 | -71.48 to 2.011 | -1.58 |
| GCL mRNA expression | 7 | 0.7799 | 12 | 0.4506 | 0.1657 | -0.2873 to 0.628 | 0.42 |
| SOD1 mRNA expression | 7 | 0.4892 | 12 | 0.6335 | -0.08143 | -0.441 to 0.281 | -0.26 |
| SOD2 mRNA expression | 8 | 0.3512 | 12 | 0.7315 | -0.06143 | -0.4425 to 0.3196 | -0.19 |
| iNOS mRNA expression | 7 | 4.5660 | 12 | 0.0006* | -0.5943 | -0.8778 to 0.3107 | -2.44 |
| Extracellular IL-6 levels | 8 | 4.5740 | 14 | 0.0004* | 30.63 | 16.26 to 44.99 | 2.29 |
| Extracellular IL-10 levels | 8 | 4.865 | 14 | 0.0003* | 34.75 | 19.43 to 50.07 | 2.43 |
| Extracellular IL-1β levels | 8 | 0.6175 | 14 | 0.5468 | -4.500 | -20.13 to 11.13 | -0.31 |
| Extracellular TNF-α levels | 8 | 0.8955 | 14 | 0.3857 | -5.250 | -17.82 to 7.324 | -0.45 |
| NLRP3 mRNA expression | 7 | 0.3240 | 12 | 0.7515 | 0.05714 | -0.3271 to 0.441 | 0.17 |

| | | | | | | | |
|---|---|---|---|---|---|---|---|
| NFκB p65 mRNA expression | 7 | 4.8250 | 12 | 0.0004* | -0.6414 | -0.9311 to 0.3518 | -2.58 |
| COX-2 mRNA expression | 7 | 3.626 | 12 | 0.0035* | -0.6400 | -1.025 to -0.2554 | -1.94 |
| Nrf2 mRNA expression | 7 | 4.6280 | 12 | 0.0006* | 1.181 | 0.6252 to 1.738 | 2.47 |
| HO-1 mRNA expression | 7 | 0.2168 | 12 | 0.8320 | 0.03714 | -0.3364 to 0.410 | 0.8320 |
| AMPK mRNA expression | 7 | 3.0692 | 12 | 0.0097* | -0.4843 | -0.8281 to -0.1405 | -1.64 |
| SIRT1 mRNA expression | 7 | 5.1782 | 12 | 0.0002* | 1.741 | 1.009 to 2.474 | 2.77 |
| PGC-1α mRNA expression | 7 | 3.668 | 12 | 0.0032* | -0.5957 | -0.9495 to -0.2419 | -1.96 |
| PGC-1α immunocontent | 6 | 1.2740 | 10 | 0.2316 | 26.59 | -19.92 to 73.09 | 0.73 |
| DCFH levels (newborn) | 6 | 0.0201 | 9 | 0.9843 | -0.4000 | -45.24 to 44.44 | -0.01 |
| GS activity (newborn) | 12 | 0.8104 | 22 | 0.4264 | -0.04750 | -0.1691 to 0.07405 | -0.33 |
| GSH levels (newborn) | 10 | 1.9490 | 18 | 0.0671 | -33.20 | -68.99 to 2.592 | -0.87 |
| Extracellular lactate levels (newborn) | 12 | 0.1566 | 22 | 0.8770 | -0.01250 | -0.1781 to 0.1531 | -0.06 |
| MTT reaction (newborn) | 9 | 0.2455 | 16 | 0.8092 | -1.622 | -15.63 to 12.38 | -0.44 |

Comparisons between the two groups (control vs. melatonin) were performed using Student's t-tests for independent samples, as the data were normally distributed (assessed by the Shapiro–Wilk test). For each parameter analyzed, the table reports the number of independent experiments (n), the t-value, degrees of freedom (df), p-value, difference between means, 95% confidence interval (CI) range, and the standardized effect size (Cohen's d), calculated using the pooled standard deviation. Asterisks indicate statistically significant differences ($p < 0.05$). "Newborn" refers to experiments performed on astrocytes obtained from neonatal rats, as described in the Materials and Methods section.
